# Dendrimers as Antiamyloid Agents

**DOI:** 10.3390/pharmaceutics14040760

**Published:** 2022-03-31

**Authors:** Svetlana A. Sorokina, Zinaida B. Shifrina

**Affiliations:** A.N. Nesmeyanov Institute of Organoelement Compounds, Russian Academy of Sciences, 28 Vavilov St., 119991 Moscow, Russia; sorokinas@ineos.ac.ru

**Keywords:** dendrimers, proteins, amyloid diseases, antiamyloid activity, molecular dynamic

## Abstract

Dendrimer–protein conjugates have significant prospects for biological applications. The complexation changes the biophysical behavior of both proteins and dendrimers. The dendrimers could influence the secondary structure of proteins, zeta-potential, distribution of charged regions on the surface, the protein–protein interactions, etc. These changes offer significant possibilities for the application of these features in nanotheranostics and biomedicine. Based on the dendrimer–protein interactions, several therapeutic applications of dendrimers have emerged. Thus, the formation of stable complexes retains the disordered proteins on the aggregation, which is especially important in neurodegenerative diseases. To clarify the origin of these properties and assess the efficiency of action, the mechanism of protein–dendrimer interaction and the nature and driving force of binding are considered in this review. The review outlines the antiamyloid activity of dendrimers and discusses the effect of dendrimer structures and external factors on their antiamyloid properties.

## 1. Introduction

Over the past two decades, the efforts of scientists in the field of dendrimer chemistry have been focused on the practical application of dendritic macromolecules. In particular, the biological application of dendrimers has received widespread scientific attention, as evidenced by a large number of publications and reviews [1,2,3,4,5,6,7,8]. The biophysical properties of dendrimers are based on their structural features, namely, precisely controlled structure, shape and sizes of macromolecules, a large number of functional groups that are capable of modification, the presence of cavities, the ability to form “guest–host” complexes, as well as the low polydispersity of macromolecules [9]. These features of dendrimers provide the possibility of fine tuning the structure to impart the necessary set of properties depending on the task. Thus, dendrimers can be used as vectors for gene delivery [2,10], targeted drug carriers [4,11] and new antiviral [12,13], antibacterial [13], anti-inflammatory [14] and antiamyloid agents [6,15,16].

At the same time, the investigation of the biological properties of new complex macromolecules should include the fundamental principles of their interaction with biological objects essentially presented in the cells. Among them are proteins which perform enzymatic, transport, building, regulatory and other functions in the organism. However, the structural conversion of proteins associated with the formation of amyloid fibrils leads to the progression of neurodegenerative diseases, which have no effective therapy so far [17,18].

The formation of stable dendrimer–protein complexes determines the ability of dendrimers to interfere with amyloid fibril formation. There are several mechanisms of antiamyloid activity depending on the dendrimer structure. The efficiency of dendrimer implementation is governed by the biophysical properties of the complexes formed, i.e., stability, binding constant, alterations in protein secondary structure, etc. These features are considered below. A fine balance between dendrimer size, number and type of functional groups is required for the proper activity correlated with low toxicity.

## 2. The Key Features of Dendrimer Structure Providing the Bioapplication

### 2.1. Dendrimer Properties

The word “dendrimer”, firstly introduced by D.A. Tomalia, consists of two Greek words which reflect the chemical structure of these molecules: *dendros* (tree) and *meros* (part). Dendrimers consist of repetitive units—monomers—growing from the central core usually in a symmetrical, radial manner [9,19]. Dendritic chemistry has been widely extended in recent decades and new types of architecture have emerged: dendrons, bow tie dendrimers, Janus dendrimers and dendrimersomes [20,21]. They could be either symmetric or asymmetric, impart blocks of different chemical composition and combine the heterofunctional units of distinctive nature and properties, but they have several distinguishing features in common. They are highly branched, have sufficiently high molecular weight and very low polydispersity index. 

Dendrimer synthesis is based on a step by step procedure in which the product is isolated, purified and characterized after each iterative step. This approach allows for effective control over the molecular weight, size, spatial structure, surface functionality, density, topological placement and chemical composition of the inner and outer parts of the molecule. Consequently, the dendrimers are widely explored in biomedicine, where tight control over the molecule structure is of particular importance.

One of the key benefits of dendrimers is their low polydispersity along with high molecular weight. Due to sequential synthesis, the dendrimer polydispersity index is M_w_/M_n_ < 1.01–1.05, which drastically distinguishes them from the other branched high-molecular-weight compounds such as hyperbranched polymers, dendrigrafts and dendronized polymers. Therefore, the dendrimers are considered as individual macromolecular compounds. The monodispersity has been demonstrated and verified by high-performance liquid chromatography, gel permeation chromatography, mass spectrometry, gel electrophoresis and transmission electron microscopy [9,22,23]. However, structural defects due to incomplete functional group substitution, intramolecular cyclization or dimer formation are possible for high-generation dendrimers. Nevertheless, the molecular weight distribution is still lower than 1.05 [19,24].

Dendrimers have nanometer sizes increasing with the generation number. For example, poly(amido amine) (PAMAM) dendrimer of the first generation is 1.1 nm in size, while the tenth generation is 12.4 nm [9,25]. The spatial structure is also adapted during the molecule growth. The low-generation PAMAM dendrimers (1–3) represent ellipsoids, whereas the shape of high-generation dendrimers is close to the spherical one. The spherical shape ensures high number and dense packing of surface functional groups. These features facilitate the dendrimer interactions with biological macromolecules of approximately similar size and shape, providing compatibility and aiding in complex stability. For that reason, dendritic structures are often considered as biomimetics. The nanometer size allows for ease of cell penetration by endocytosis pathways and the ability to cross the blood–brain barrier (BBB).

Dendrimers exhibit high solubility that essentially simplifies their characterization. The presence of functional groups such as carboxyl, hydroxyl and amino groups in the molecule periphery improves the solubility in polar solvents. There are three domains in dendritic architecture tentatively distinguished in the molecule: the core, interior and surface possessing reactive groups exposed in the solvent. Each structural domain has a specific function relevant to biomedical applications. The core defines the degree and dimensions of branching. In case of high-generation dendrimers with a high branching degree and spherical shape, dense packing of surface functional groups is observed. As a result, a specific microenvironment, usually hydrophobic, along with cavities is formed in the interior part. This provides the possibilities of physical entrapment of small molecules with the formation of host–guest complexes. This feature is usually utilized for encapsulation of lipophilic drug molecules with the formation of drug delivery systems based on physical interactions of therapeutic agents with the internal structure of the dendrimers.

The surface functional groups offer ample opportunities for their direct modification in order to impart the desired properties or to conjugate with different molecules. This outer layer performs several functions. The terminal functional groups define the interactions with biomolecules, particularly the mechanism, the complex stability, etc. [10,26,27,28]. They could be modified in order to reduce toxicity, to insert the sensitive groups or to improve the targeting properties [7,29]. For example, the conjugation with monoclonal antibodies significantly enhances the selectivity of interaction with target proteins [30], while decoration with folic acid increases the affinity to cancer cells [31,32]. Finally, the terminal groups can be employed for chemical conjugation with drug molecules [4,33]. The main advantage of this approach is the ability to bear multiple copies of a drug in a single carrier. In this case, the use of cleavable (under certain conditions) linkers between dendrimer and drug molecules is preferable. 

However, the most important feature of dendrimers inspiring their biological application is the multivalency. The structural domains together with a variety of functional groups allow one to combine several different entities in a single molecule, making the dendrimer structure adaptable for a desired purpose. The dendrimers could possess the inherent therapeutic properties, e.g., antiviral, antibacterial or antiamyloid, along with enhanced specificity to target molecules [10,11,13]. They could address the issues of the construction of multidrug systems bearing structurally different therapeutic agents such as siRNA, cytostatics and peptide/protein for antitumor therapy [2,3,5,20,34,35]. In other words, the dendrimers represent a perfectly described nanoplatform for implementation of desired functions in a prescribed manner.

### 2.2. Approaches to Decrease Toxicity and Improve Biocompatibility 

The main requirements for synthetic molecules intended for biomedical application are low toxicity, non-immunogenicity, and good biocompatibility. A large number of publications have dealt with the study of toxicity of various classes of dendrimers both in vitro and in vivo [5,7,36,37]. 

Early studies have shown that dendrimer cytotoxicity is associated with the disturbance of cell membrane integrity due to tight interaction of spherical dendrimers with a dense arrangement of terminal groups. This promotes the hole formation, leakage of cellular content and cell death. Later, it was found that such a cytotoxicity mechanism is observed mainly for high-generation dendrimers with large size and positive charge. Nowadays, the established cytotoxicity mechanism is attributed to the dendrimer localization at the mitochondria and damage of the mitochondrial membrane. This leads to the appearance of reactive oxygen species (ROS) that cause oxidative stress and DNA damage, disruption of the mitochondrial electron transport chain and oxidative phosphorylation processes, inhibition of cytochrome C oxidase activity and nicotinamide adenine dinucleotide phosphate (NADPH) synthesis [38,39,40,41,42].

The cytotoxicity of dendrimers depends on their chemical composition, size, nature of terminal groups and backbone [7,37]. The dendrimers demonstrate the generation-dependent toxic effect. In general, high-generation dendrimers possess greater toxicity than low-generation ones. Nevertheless, a study by Fischer [43] showed that PAMAM dendrimers exhibited lower toxicity compared to the linear polymer of analogous chemical structure. At the same time, cationic PAMAM and poly(propylene imine) (PPI) dendrimers have approximately the same cytotoxic effect [44]. A comparative analysis of the toxicity of cationic (terminal amino groups) and anionic (terminal carboxyl groups) dendrimers showed a significantly lower toxicity of anionic compounds [45]. Moreover, low-generation PAMAM dendrimers with carboxyl terminal groups have neither cytotoxic nor hematotoxic effects up to a concentration of 0.35 mM [46].

There are several established approaches affecting the dendrimer cytotoxicity. The first one is based on the surface modification which masks the cationic charge. Pegylation, acetylation, lipid and carbohydrate modification, fluorination and other techniques are widely explored to reduce toxicity. Thus, modification of PAMAM terminal amino groups with PEG increased the LD50 value to 1 mM versus 0.13 mM of the parent dendrimer for the Caco-2 cell line [47]. Similar dependences were also found in other works [48,49]. The pegylation of PPI dendrimers also reduced their toxicity [37,50]. A decrease in nitrogen basicity affects the cell viability, ranking nitrogen quaternization as a promising method for reducing toxicity [51,52]. Conjugation of dendrimers with dexamethasone [53], phenylalanine [54], porphyrin [55], arginine [56,57], esterification of surface groups [58] and backbone [59] sufficiently suppresses toxicity.

Fluorination has emerged as a promising tool improving the stability, biocompatibility and pharmacokinetic behavior of drug molecules [60,61]. The fluorinated compounds preserve the ability to penetrate the cells and might even enhance the cellular uptake [10]. Thus, the PAMAM dendrimers modified with perfluoroalkyl moieties demonstrated increased cellular uptake and endosomal escape with decreased toxicity for a list of cell lines [62]. The same trend was observed for PPI dendrimers [63].

Another versatile strategy for improving biocompatibility is the incorporation of lipid moieties. This modification supplies the neutral charge and increases the affinity to the cell membrane since the lipids are natural constituents of the membrane bilayer. PAMAM dendrimers conjugated with fatty acids of different lengths demonstrated an enhanced cell penetration ability [64]. C10-alkane-modified PPI dendrimer showed excellent cellular uptake without apparent toxicity [65]. Another study revealed that C18-alkane-modified poly-_L_-lysine dendrimer possesses improved biocompatibility [66]. Simultaneous presentation of lipid and PEG groups in a dendrimer molecule allows for the generation of micelles in solution, which are now widely explored as non-toxic, non-immunogenic and highly effective cargo for drug delivery [67].

### 2.3. Permeability of Blood–Brain Barrier

The treatment of many diseases of the central nervous system is complicated by the presence of the blood–brain barrier (BBB). The BBB is a dense layer of endothelial cells with a pore size of less than 1 nm, which separates the nervous tissue from the bloodstream and selectively passes only the necessary nutrients and bioactive substances.

As a rule, passive transport of medicinal substances is possible for compounds with a molecular weight M_w_ < 400 Da and able to form less than seven hydrogen bonds, or with a sum of nitrogen and oxygen atoms in a molecule not exceeding five [68]. However, recent studies have shown that various nanometer-sized particles, including liposomes, polymeric nanoparticles, lipid micro- and nanoparticles, polymeric nanogels and polymeric micelles are able to penetrate the BBB [6,69]. One of the proposed entry mechanisms is receptor-mediated endocytosis. In particular, some commercial technologies, such as the Trojan Horse, use specific endogenous ligands to bind the receptors on the surface of capillary cells [68,70].

A number of studies proved dendrimers’ ability to cross the BBB, presumably via above-mentioned absorption-mediated endocytosis [11,71,72,73,74]. In [75], PAMAM dendrimers modified with PEG bound with transferritin and bearing doxorubicin in the internal cavities have been shown to efficiently cross the BBB, delivering 13.5% of doxorubicin for 2 h, while only 5% of the compound was observed for unbound doxorubicin. The size of the dendrimer conjugate varied from 14 to 20 nm. In general, molecules with sizes up to 20 nm are recognized to cross the BBB. The method of administration of the substance is important as well. Thus, many drugs can enter the brain if administered intranasally [6,74]. It should also be noted that the violation of the BBB, which is characteristic of a number of neurodegenerative diseases, facilitates the penetration of drugs from the blood into the nervous tissues.

## 3. Dendrimer–Protein Interactions

Proteins perform vital functions in the cell and in the whole organism. They participate in the transport of substances across the cell membrane, acting as receptors and structural units. The existence of enzymes ensures the passage of biochemical reactions. The drug administration, pharmacokinetic and pharmacodynamic processes are aligned with protein–drug interactions since the plasma contains more than 2400 proteins [76,77].

Given the potential biomedical applications of dendrimers, the knowledge of their protein-binding properties, the driving force and mechanism of interactions, composition and characteristics of the formed complexes, dependence of structural and morphological changes in proteins on the dendrimer molecular characteristics is of particular importance.

### 3.1. Nature of Protein–Dendrimer Interactions

The nature of dendrimer–protein interactions has been investigated in numerous publications [27,78,79,80,81,82,83,84,85,86,87]. In general, the binding of dendrimers with proteins is driven by the electrostatic interactions between dendrimers and oppositely charged amino acid residues. However, other forces are also involved in complex formation. These are the hydrophobic interactions of non-polar groups of dendrimers and hydrophobic parts of amino acids, hydrogen bonds and van der Waals interactions. The contribution of each type of interaction strongly depends on the dendrimer characteristics.

The charged cationic and anionic dendrimers are generally believed to interact with proteins through electrostatic forces [27,79,88], while neutral dendrimers tend to form complexes through hydrophobic interactions and hydrogen bonds, as was exemplified by PAMAM-OH and PPI dendrimers modified with terminal maltose groups [89,90,91]. Nevertheless, some authors report that hydrophobic forces and hydrogen bonding are driving forces for interaction even for charged dendrimers. In [80], the hydrophobicity has been determined to play a major role in interactions between dendrimers with various terminal groups (cationic primary amine group, anionic sulfate group, a highly hydrophobic benzoate group, and the monosaccharide α-D-mannose) with intrinsically disordered protein NUPR1. However, molecular dynamic (MD) simulations point out that hydrogen bonds and electrostatic forces played a further key role, modulating the hydrophobic interactions. In another work [92], hydrophilic, H-bonding and van der Waals interactions have been recognized as the driving forces of the PAMAM G4.5 interaction with trypsin and trypsin inhibitor. 

It should be noted that electrostatic interactions are also observed for similarly charged dendrimers and protein. The protein charge determined by zeta-potential measurements corresponds to the net charge of the molecule. In fact, the heterogeneity of charge localization always exists. Although the net charge of the molecule could be positive, there are oppositely charged domains, which offer the opportunity for complexation with polycations, and vice versa. This behavior has been previously reported for interactions of similarly charged proteins and linear polyelectrolytes [93,94,95]. This phenomenon was investigated in more detail in [81]. The authors determined that PAMAM G5.5 dendrimers and BSA possessing the positive charge at pH 5.0 interact through electrostatic forces due to the negatively charged structural domain I of BSA. At physiological pH 7.5, both BSA and dendrimers have a negative charge. In this case, binding occurs via interaction of dendrimers with positively charged domain III. In our work [82], we demonstrated an effective interaction of cationic pyridylphenylene dendrimers with positively charged prion protein. The complex formation proceeds effectively with high binding constants between positively charged dendrimers and negatively charged regions of the protein surface, as was determined by MD simulations.

**Figure 1:**
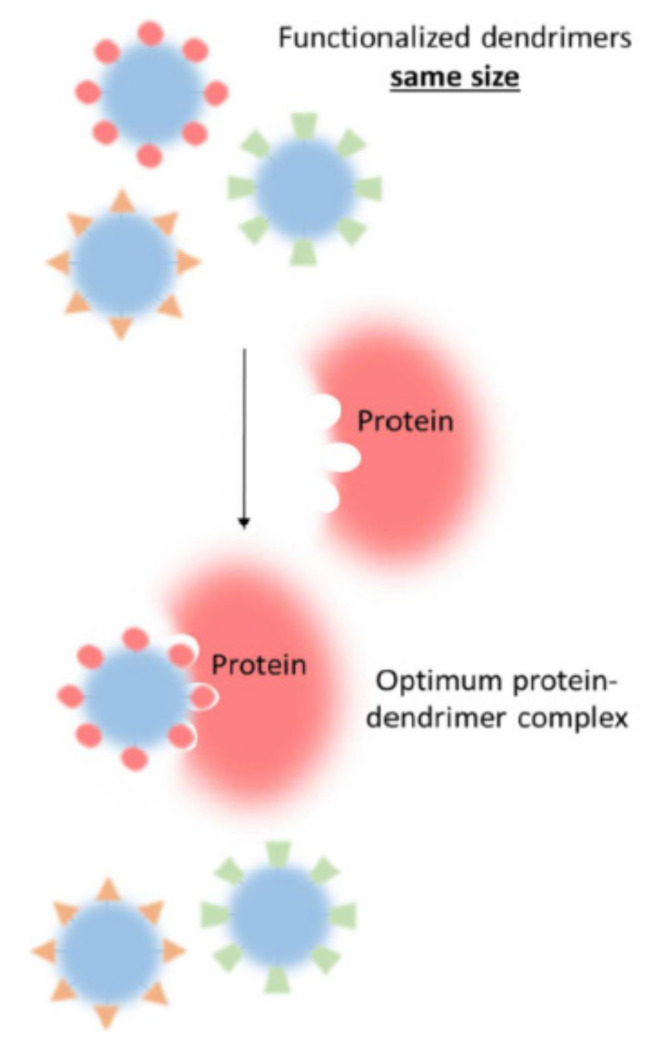
Schematic representation showing how terminal functionality is important with respect to optimum protein binding and selectivity. Reprinted with permission from ref. [28]. Copyright 2017, American Chemical Society.

Modification of terminal groups has a great impact on the mechanism of interaction with proteins. A schematic representation of the influence of dendrimer terminal groups on binding is given in Figure 1. If the dendrimer surface preserves the charge after modification, the electrostatic forces are responsible for complexation. Modification with neutral groups alters the driving force of interaction according to the nature of the surface groups. For example, attachment of the neutral maltose residues to PPI dendrimers changes the mechanism of interaction with prion protein from electrostatic to H-bonding [90]. The surface functionalization may either strengthen or weaken the association. The introduction of guanidine groups into the PAMAM dendrimer structure enhanced the binding with the protein due to an increase in the number of charged groups involved in complexation [96].

Nevertheless, in general, the complex formation is determined by a combination of molecular interactions. The contribution of each type of interaction may be assessed through a careful evaluation of thermodynamic parameters of binding, which could be derived from the results of isothermal titration calorimetry (ITC) experiments along with computational methods [80,81,82,92].

The dendrimer–protein complex stability is another important factor determining the potential application of dendrimer–protein interactions in nanomedicine. Tentatively, this feature results from high affinity of dendrimers to proteins and high values of association constants arising from multipoint contacts. Nevertheless, the mechanism of interaction and driving force are also of particular importance for complex stability. Thus, the hydrophobic interactions have been shown to play a crucial role in the resistance of complexes toward disruptive action of competitive compounds and aid in the complex stability under physiological conditions (0.14 M NaCl). In [97], the cooperation of electrostatic and hydrophobic interaction has been shown to improve the stability of polyion complexes. The presence of aromatic groups, together with the carboxylate ones at the terminal functionalities of the dendrimer, allowed a more specific and favorable binding of dendrimers with NUPR protein [80]. The hydrophobic interactions are responsible for the stability of the complexes toward the dissociative action of low-molecular-weight electrolytes such as sodium chloride. While electrostatic interactions are known to become weaker under high ionic strength, the hydrophobic forces ensure the complex stability under a wide range of ionic strength [82]. The hydrophobic forces contribute to the insusceptibility of the dendrimer–protein complexes to competitive interactions with charged polyelectrolytes.

### 3.2. Influence on the Protein Secondary Structure

Binding of dendrimers may cause changes in the protein secondary structure, which could be evident from circular dichroism (CD) measurements. In most cases, a decrease in α-helix content accompanied by a slight increase in β-sheets and random coil structure is observed [81,83,84,87,92]. The dendrimers may also induce the alteration of tertiary and quaternary structures of well-folded proteins [84] or partial protein destabilization [92].

The influence of dendrimers on the secondary structure of proteins strongly depends on the dendrimer/protein molar ratio. The electrostatically driven binding of a hybrid carbosilane–viologen–phosphorous dendrimer with human serum albumin (HSA) did not induce conformational changes in protein molecules up to 2-fold molar excess of dendrimers, while the addition 5- and 10-fold molar excess significantly altered the shape of albumin CD spectrum [87]. The same trend was observed in other studies, indicating the necessity of large dendrimer excess to induce the changes in protein secondary structure [81,82].

Besides the dendrimer/protein ratio, the ability to trigger the protein conformational changes is also governed by the dendrimer structure and nature of terminal groups, thus determining the mechanism and efficacy of interaction. The changes in dendrimer structure vary their impact on protein molecules. For example, PAMAM dendrimers of the third and fifth generations substituted with sugar residues did not alter the secondary structure of albumin, while PEG-modified cationic PAMAM dendrimers showed strong binding to albumin, affecting its secondary structure and conformation [98,99]. The effect of PPI dendrimers on insulin also depended on the nature of the end groups of the dendrimer (unmodified or modified with guanidine or urea) [100]. However, all dendrimers changed the secondary structure and thermal stability of the protein. Thus, the nature of the terminal groups of the dendrimer plays an important role in their interaction with proteins.

In general, the conformational changes in protein occur in case of tight binding accompanied with high values of association constants, which are usually characteristic of electrostatically driven interactions [84]. In [87], the authors revealed the pronounced changes in HSA secondary structure upon the interaction with carbosilane–viologen–phosphorous dendrimers bearing 12 inner and 24 outer charges and interacting with high-binding constants even at low dendrimer concentration, while no conformational changes were observed for HSA interaction with the similar dendrimer possessing 24 total positive charges and weaker binding constant at the same dendrimer concentration. The results suggest that alteration in protein structure is a consequence of high strength and affinity of interaction.

Quantitatively, the strength of an interaction is described by the association constant. The association constant can be calculated by different experimental techniques. The most widely used are ITC and the fluorescence quenching method. The fluorescence quenching method is based on the presence of tryptophan residues in protein molecules which are extremely sensitive fluorophores [101]. Upon the complex formation, the intrinsic fluorescence is quenched due to interaction with a ligand. The construction of the plot of relative fluorescence intensity on the concentration of the ligand allows one to estimate the efficacy of binding as a Stern–Volmer constant. To exclude the unspecific decrease in fluorescence intensity due to optical properties of the sample, one should consider the possibility of overlapping absorbance spectra of protein and ligand, which should be carefully checked prior to measurements. In ITC experiments, the association constant is determined based on thermodynamic parameters derived from binding isotherm. However, an accurate determination of a binding constant is very comprehensive and requires implementation of a complex binding model since the heat released during the calorimetric titration of a protein by a ligand is a sum of different processes including protein–ligand binding, protein–protein interactions, conformational rearrangements in protein structure, etc.

The affinity of interaction assessed based on binding constant increases with an increase in the number of groups involved in complex formation. In most cases, this correlates with the dendrimer generation number and the affinity of interaction increases with the generation [80,81,82,87]. Functionalities at the dendrimer periphery also modulate the affinity for proteins and could be a valuable tool for controllable tuning of the complex properties [28].

However, the ability of dendrimers to alter the protein conformation is a basis for a valuable inherent property of dendrimers—antiamyloid activity [78]. Thus, the dendrimers can interfere with the ordered beta-sheet structure formation. Briefly, the addition of the dendrimers to amyloidogenic, usually intrinsically disordered proteins leads to formation of stable complexes which are not prone to aggregation. Despite the minor unfolding of protein structure, the tight interactions with dendrimer stabilize the protein molecule and retard the amyloid fibril formation. The antiamyloid properties of dendrimers are discussed in more detail below.

### 3.3. Computer Simulations of Protein–Dendrimer Interactions

Molecular dynamic (MD) simulations are widely used to study protein–dendrimer interactions [26,64,82,86,102,103,104,105]. This is a valuable technique supporting the experimental results and providing data which cannot be obtained experimentally. MD helps to identify the binding sites, number and location of amino acid residues involved in association; predict the complex structure and conformational changes of protein; and assess the contribution of different molecular interactions in complex formations. MD simulations are usually applied simultaneously with biophysical techniques, providing a complementary view on the complexation details.

For example, computer simulations of HSA interactions with PAMAM dendrimers revealed the participation of protons of secondary amino groups located in the inner sphere of dendrimer together with terminal amino groups [106]. This could be explained by the flexibility of dendrimer structural units able to adopt different conformations, which offers possibilities for interaction of inner groups. Computer simulations demonstrated two important impacts of PAMAM dendrimers binding with alpha-chymotrypsinogen A: the interaction enhances the conformational stability of protein and diminishes the protein–protein interactions due to wrapping by dendrimer molecules [107].

**Figure 2:**
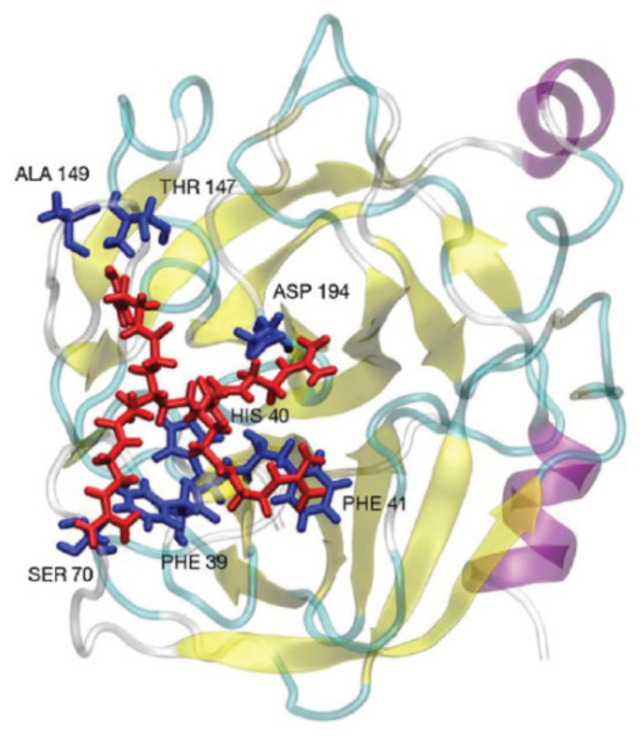
The structure of the complex between PAMAM dendrimers modified with guanidine moieties and alpha-chymotrypsinogen A obtained by molecular dynamic simulations. PAMAM dendrimer with guanidinium chloride surface interacting with multiple groups on the surface of protein. Reprinted with permission from [108]. Copyright 2011, Public Library of Science.

MD simulations were used to study the influence of dendrimer counterions on the interaction with proteins [108,109]. The study utilized PAMAM dendrimers modified with guanidine moieties bearing Cl^−^, SO_4_^2−^ and H_2_PO_4_^−^ groups as counterions and alpha-chymotrypsinogen A (Figure 2). The interaction efficiency was measured by the preferential interaction coefficient G, which is associated with the free energy of protein transfer from water to an aqueous salt solution and can be used to assess the affinity of the interaction of salt ions with the protein surface. Upon 0.18 M concentration, coefficient G equaled 1.0, 2.7 and 2.3 for Cl^−^, SO_4_^2−^ and H_2_PO_4_^−^, correspondingly. This means that Cl^−^ anions promote the dendrimers’ interaction with the protein, while SO_4_^2−^ and H_2_PO_4_^−^ inhibit binding. For Cl-containing dendrimers, the interaction proceeds through hydrogen bonding of guanidine groups of dendrimer with negatively charged amino acids of protein along with cation–π interaction with aromatic amino acids. The simulation data also revealed the possibility of simultaneous cooperative interaction of several guanidine groups with the protein surface. This results in higher strength of contacts in comparison with single functional group binding. However, switching the counterion to either sulfate or phosphate inhibits the appearance of such multiple interactions.

In another work [110], MD allows one to explore the effect of prevention of the cytokine response mediated by the TLR4-MD-2-lipopolysaccharide complex due to dendrimer binding with lymphocyte antigen MD2. The authors highlighted the importance of cooperative electrostatic interactions of dendrimer glucosamine moieties with the amino acid residues of the MD-2 protein located near the hydrophobic pocket.

**Figure 3:**
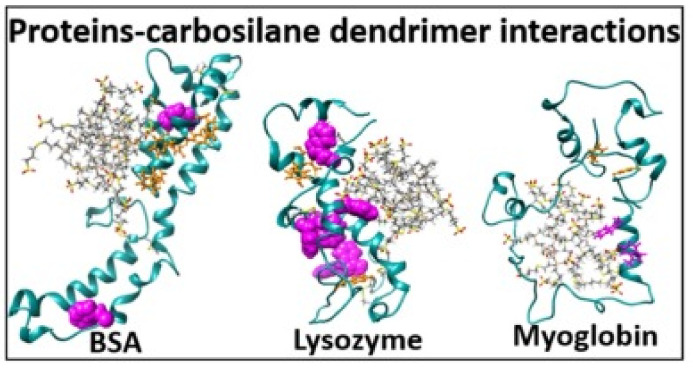
Structures of the complexes between sulphonate-terminated carbosilane dendrimers and corresponded protein obtained by MD. Reprinted with permission from ref. [88]. Copyright 2017, Elsevier.

Computer simulations were applied to investigate the interactions of sulfonated carbosilane dendrimers with three different proteins (bovine serum albumin, myoglobin, lysozyme) [88]. The study revealed the effective complex formation between negatively charged dendrimers and BSA possessing net negative charge. As it was mentioned earlier, the electrostatic interactions occurred due to the existence of positively charged regions on the protein surface (Figure 3).

## 4. Dendrimers and Neurodegenerative Disorders

### 4.1. An account of Amyloid Diseases

One of the most noticeable physiological activities of dendrimers is driven by their ability to influence the processes of protein aggregation and prevent the formation of amyloid structures. Amyloids are insoluble protein aggregates deposited in intracellular (neurofibrillary tangles) and extracellular (senile plaques) space. Currently, the ability to form amyloids is considered a natural feature of proteins [111]. Recent studies have shown that the amyloid state of proteins is used by some organisms to create the unique structures performing various useful functions [112].

However, the structural conversion of proteins associated with the formation of amyloid fibrils in humans is responsible for the onset and progression of neurodegenerative diseases, which have no effective treatment so far. Alzheimer’s [113] and Parkinson’s [114] diseases, prion diseases [115], also called transmissible spongiform encephalopathies, and some others [17] are not the total list of neurodegenerative disorders affecting humans. In general, neurodegenerative diseases are caused by the loss of the biological activity of the protein due to its unfolding and aggregation into amyloid fibrils [17,116,117].

The factors affecting the protein unfolding and driving the loss of native conformation are not thoroughly understood. The amino acid sequence, certain mutations, high or low pH, post-translational modifications, elevated temperature, glycation and oxidative stress are known to influence the process [118].

The formation of amyloid fibrils is a consecutive process [117,119]. It includes the unfolding of the native protein, followed by the formation of small protein oligomers consisting of 10–40 molecules of monomeric proteins, further association of oligomers into fibril nuclei and, at the final stage, formation of mature amyloid fibrils saturated with a beta-sheet structure [120,121,122]. At the same time, amyloidogenic proteins (alpha-synuclein, beta-amyloid peptide, prion protein, etc.) may be structurally and functionally distinct, but the structure and properties of their fibrils are very similar [123,124].

Amyloid fibrils are long, unbranched structures with a diameter of 6–12 nm [122]. The beta-strands in these structures are oriented perpendicular to the fibril axis and stabilized by hydrogen bonds parallel to the fibril axis. The aggregation process is accompanied by the alteration in protein secondary structure. While the native conformation of a protein is mostly presented by alpha structure or a random coil, the oligomers demonstrate a significant increase in beta sheets content, the contribution of which becomes even more pronounced upon the transition into the amyloid fibrils state [112,122,125]. The most recent studies report that metastable oligomers and protofibrils, rather than mature amyloid fibrils, possess the highest toxicity [126,127,128].

**Figure 4:**
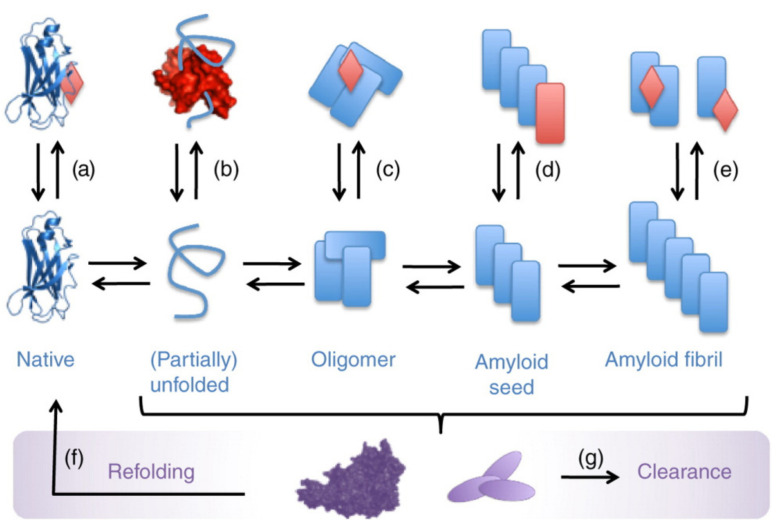
Schematic illustration of strategies to inhibit the formation of amyloid fibrils. (a–e) Biochemical mechanisms of action that could be employed by ligands (red) binding to different states of amyloid-forming peptides (blue). (a) Stabilization of the native state. (b) Sequestering of monomeric peptide. (c) Stabilization or promotion of off-pathway oligomers. (d) β-Sheet breakers that terminate fibril elongation. (e) Disassembly of amyloid fibrils. In addition, refolding (f) and clearance (g) mechanisms (purple) can be important in vivo. Reprinted with permission from ref. [119]. Copyright 2012, Elsevier.

Considering the sequential character of the fibril formation process, there are several opportunities for intervention (Figure 4) [119]. The compounds might bind the protein in a monomeric state and stabilize its structure, retaining protein from the aggregation. The ligands might also bind the protein oligomers or termini of the growing fibrils, thus preventing the aggregation. Alternatively, the dissolution of existed aggregates might be another approach to influence the amyloidosis. Importantly, the formed complexes should be stable and not prone to re-aggregation and should not serve as a source of new toxic oligomeric species. The strong association of the ligands with protein greatly contributes to that purpose.

### 4.2. Dendrimers Prevent Amyloid Fibril Formation and Dissolve Amyloid Aggregates

The antiamyloid activity of dendrimers includes two main strategies of intervention: the inhibition of amyloid fibril formation and dissociation of formed aggregates. The dendritic shape ensures a plenty of functional groups located on the surface and, accordingly, a large charge and charge density. As it was mentioned, this feature determines the formation of tight complexes with the protein due to multivalent interactions, which is beneficial for the stabilization of the protein molecule.

Certain advances in this field were achieved using low-molecular-weight chemical compounds [129], such as phenol [130,131], curcumin [132,133] and cinnamic acid [134,135] derivatives, and some polymers [136,137,138,139], e.g., polystyrene sulfonate [140,141]. However, the use of low-molecular-weight compounds is not effective due to their weak binding with protein. Moreover, the abilities of small molecules to bind the most toxic prefibrillar forms of amyloid proteins and reduce their toxicity are very limited. This is related to the difficulty of targeting of relatively large, solvent exposed, flat protein surfaces. The polydispersity of synthetic polymers complicates the investigation of the resulting protein–polymer complexes due to their nonuniformity. Furthermore, long and short polymer chains often produce different and sometimes even opposite effects: the long chains can inactivate the protein, while the short chains, in contrast, can promote aggregation. Therefore, the use of polymers does not afford unambiguous and reproducible results.

However, dendrimers are probably the most promising compounds which were shown to possess antiamyloid activity. As it will be shown below, the peculiarities of dendritic structure, such as branching, monodispersity and multivalency, have been proven to be advantageous for antiamyloid activity. The up-to-date studies consider two main possibilities to modulate the neuropathy (inhibition of aggregation and disruption of aggregates) of various dendritic structures. 

The first studies of the antiamyloid activity of dendrimers were carried out using four generations of PAMAM dendrimers and second and fourth generations of PPI dendrimers [142,143]. Studies have been conducted on scrapie-infected neuroblastoma cell lines. Scrapy is a disease of sheep caused by a disturbance of the secondary structure of the prion protein, which changes its normal form (PrP^C^) to an infectious (PrP^Sc^) one, saturated with beta-sheets. It turned out that dendrimers are able to remove PrP^Sc^ from neuroblastoma cells, with the efficiency increasing with generation number. Obviously, the antiamyloid activity of dendrimers is a function of large charge and charge density. This was confirmed by the absence of activity of neutral PAMAM-OH dendrimers, as well as linear polymer analogues. The result also depended on the concentration of the dendrimer and the duration of the experiment. Noticeably, PrP^Sc^ was not detected in the three weeks after removal of the dendrimer from the medium. The authors found that after treatment with dendrimers, PrP^Sc^ becomes susceptible to proteolysis (which is typical for the normal cellular form of the prion), while the infectious form is characterized by resistance to the action of protease K.

Phosphorus-containing dendrimers have been also shown to be promising anti-prion agents [144,145]. They effectively inhibited the formation of PrP^Sc^ in neuroblastoma cells [144]. However, the authors found that a larger dendrimer charge does not necessarily lead to an increase in efficiency. The fourth generation was more effective than the third, while the fifth had a less pronounced effect compared to the fourth. This suggests that an optimal balance between the size and the number of surface functional groups is required for compounds to demonstrate the highest activity.

In vitro experiments confirmed the ability of dendrimers to influence and prevent the process of amyloid protein aggregation. The results indicate the crucial role of dendritic structure, namely, the large charge and its high density, in antiamyloid activity. Dendrimers exhibited antiamyloid properties in contrast to linear analogues of the same molecular weight and charge [146]. PPI dendrimers modified with guanidine groups suppressed fibrilization of the prion peptide 106–126 much more efficiently than unmodified ones [96]. The effect was explained by the excess of charge of guanidine-modified dendrimers. 

In contrast to in vivo experiments, neutral dendrimers demonstrated the ability to destroy amyloid aggregates in vitro, as it was exemplified by PPI dendrimers modified with maltose groups [89]. At the same time, a similar dendrimer with partially substituted maltose groups by PEG with terminal amino groups had no effect on protein aggregates [90]. In this case, PEG acted as a flexible spacer, reducing the density of surface functional groups.

The ability of the most commonly used amine-containing dendrimers to suppress protein propagation strongly depends on pH. The effect is attributed to the high degree of protonation of both dendrimer amino groups and protein histidine residues achievable at low pH. Thus, the most favorable conditions for the complex formation are in the range of low pH values [90,142,147]. For example, PEI and PPI dendrimers, which effectively removed PrP^Sc^ from neuroblastoma cells at pH = 4.0, had no effect under neutral conditions [142,143].

Another important feature influencing the antiamyloid behavior of flexible dendrimers, such as PAMAM, PPI, PEI, carbosilane and others, is the tendency to back-fold. This implies the folding of dendrimer chains toward the center of the molecule. As a result, the number of functional surface groups accessible for interaction with the protein as well as the topology of the dendrimer molecule are sufficiently affected [90,148,149,150,151].

Noticeably, the application of cationic dendrimers has been demonstrated to be more advantageous for anti-prion properties. While the fifth generation of anionic PPI dendrimers modified with sulfogroups efficiently removes PrP^Sc^ in vitro, the studies on cell lines revealed no activity [90]. This was explained by the poor permeability of negatively charged cell membranes for similarly charged anionic dendrimers.

**Figure 5:**
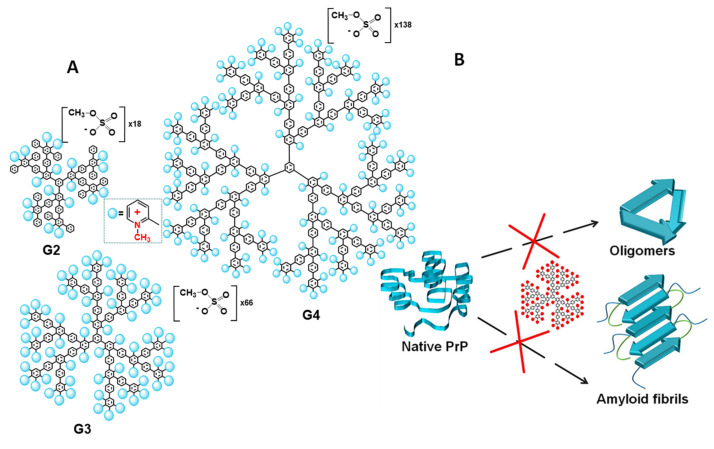
Structures of CPPDs of the second, third and fourth generations (**A**) and schematic representation of CPPDs influence of amyloidogenic prion protein (**B**).

Alternatively, to flexible dendrimers, there are rigid dendrimers with constant arrangement of functional groups on the surface, invariable spatial structure and topology of the molecule. In our work [52], the rigid cationic pyridylphenylene dendrimers (CPPD) with permanent charge and charge density were used to modulate the prion propagation in vitro *(*Figure 5A). The dendrimers were found to inhibit the formation of not only amyloid fibrils but also amyloid oligomers, which are currently believed to be the most toxic amyloidogenic forms of proteins [152,153,154,155] (Figure 5B). The addition of CPPD to the prion protein during the specially launched formation of the oligomers blocked its structural conversion and prevented aggregation. The structures obtained in the presence of the dendrimers displayed essentially lower content of beta-sheets compared to the control oligomers, as was confirmed by the results of CD spectroscopy. The most remarkable experiments were carried out on the neuroblastoma cell line, which clearly indicated the efficiency of the dendrimers. While control prion oligomers induced an intense decrease in cell viability measured by MTT assay, the structures obtained in the presence of the dendrimers did not affect the viability of neuroblastoma cells. The effect depended on the charge of the dendrimer molecule, which increased with the generation growth. The fourth-generation dendrimer exhibited the highest activity and suppressed the unfolding and aggregation of the prion protein at the concentration of 0.25 μM. Hence, the dendrimers not only inhibited the aggregation but also prevented the formation of toxic structures.

CPPDs further suppress a deeper aggregation, namely, the formation of amyloid fibrils. Unlike the prion amyloid fibrils which are resistant to proteolysis, the addition of the dendrimers rendered prion susceptible to proteinase K digestion. Therefore, besides the inherent ability of dendrimers to prevent amyloid aggregation, other cell mechanisms, such as protease digestion, can support the dendrimer antiamyloid action in vivo. Moreover, due to the strong association with the dendrimers, the resulting prion protein–dendrimer complexes did not exhibit infectivity, e.g., the ability to induce the amyloid transformation of cellular PrP, which is characteristic of unfolded forms (unfolded monomers, oligomers, and protofibrils) of PrP^Sc^. In other words, the prion protein loses the amyloidogenic properties in the complex with dendrimers.

**Figure 6:**
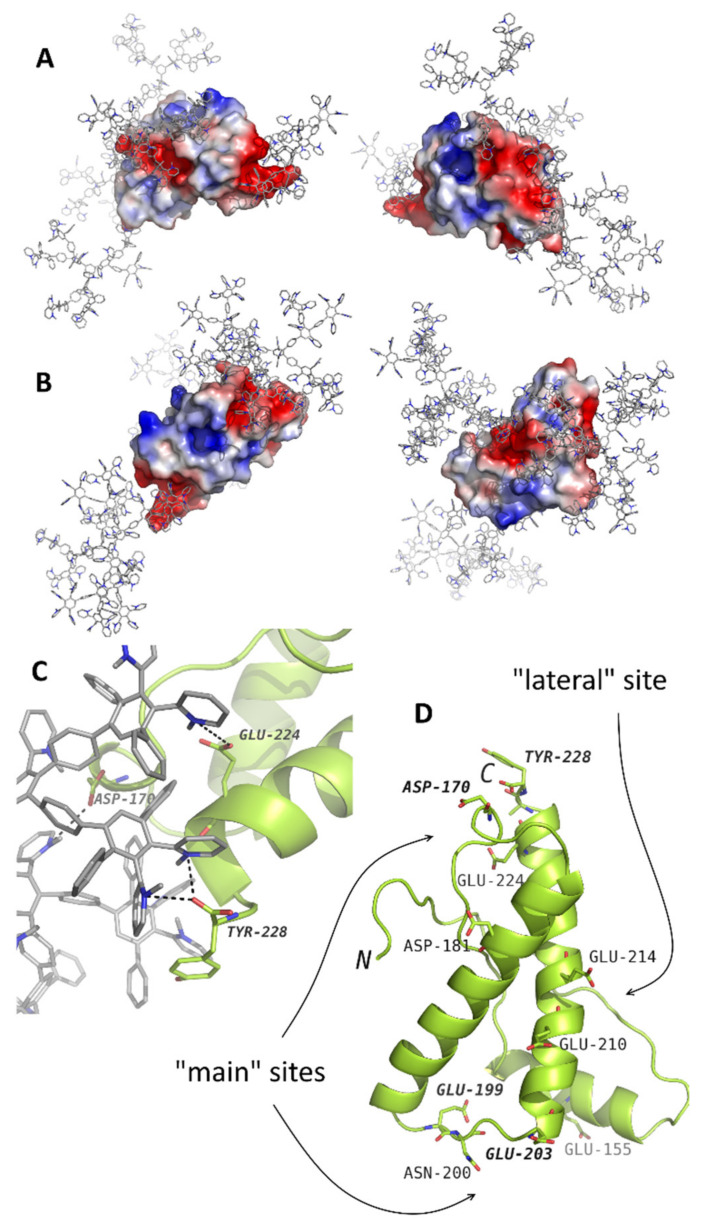
The results of molecular dynamics simulations of prion protein (the structure from the PDB entry 1tqb) with CPPDs of the second (**A**) and third (**B**) generations. The typical binding positions are shown. Dendrimers are shown in gray with blue nitrogen; the protein is colored according to electrostatic potential of the surface, where positively charged regions are blue, and negatively charged regions are red. The panel (**C**) represents an example of interactions in detail. The dendrimer-binding residues are shown in the panel (**D**). Reprinted with permission from ref. [82]. Copyright 2017, Royal Society of Chemistry.

Obviously, the binding sites are of particular importance for the antiamyloid properties of compounds. The MD simulations of CPPD interaction with the full-length prion protein demonstrates that the binding of these dendrimers affects the region 190–200, which is involved in the amyloid transformation of the protein [82] (Figure 6). The hydrophobic interactions and multipoint contacts enable strong binding and do not allow the complex to dissociate, which affords the reliable stabilization of the protein by the dendrimer. The aggregation inhibition occurs at the neutral pH values, whereas the polyamine compounds explored earlier were active only at low pH values [146].

CPPD were also active in the destruction of prion amyloid aggregates. The addition of CPPD to insoluble amyloid aggregates of the prion protein led to their decomposition due to the formation of complexes with the dendrimer and conversion of the resulting complexes to a soluble state [156]. The dendrimers preserved the activity at the physiological pH = 7.4. The resulting complexes were stable and did not undergo re-aggregation owing to the hydrophobic interactions observed earlier.

The investigation of antiamyloid activity of dendrimers was further extended to other amyloidogenec proteins, such as beta-amyloid (Aβ) peptide associated with the onset of Alzheimer’s disease [15,145,157,158,159,160,161,162,163], alpha-synuclein involved in Parkinson’s disease [145,164,165,166,167], tau protein associated with both Alzheimer’s and Parkinson’s diseases [146] and transthyretin [168], which induced the amyloidogenic transthyretin (ATTR) amyloidosis. A wide range of dendritic compounds have been shown to preserve antiamyloid activity towards different types of proteins and peptides: PAMAM [159,161,166,168], PPI and their modified derivatives [157,158], phosphorous [145,163], carbosilane [165,167] and other types of dendrimers [160,162,164].

The dendrimers demonstrated high activity in both antiamyloid strategies: (i) inhibition of amyloid aggregation, which could be achieved due to stabilization of monomeric protein state or by blocking the ends of growing prefibrillar structures, and (ii) dissolution of misfolded toxic forms and fibrils of the proteins. The antiamyloid effect was a consequence of dendrimer interactions with amyloidogenic proteins proceeding with high affinity and following the similar rules as dendrimer interactions with other proteins. In this case, the aspects of dendrimers’ influence on protein secondary structure, the location of binding sites, the values of association constants, the consistent of dendrimer and protein topologies, the multipoint contacts and hydrophobic interactions are of great importance for high activity.

The studies revealed that the main structure–activity relationships observed previously for interaction with PrP did not change upon the protein variation. Thus, PAMAM dendrimers demonstrated excellent potential toward the prevention of Aβ propagation with activity strictly dependent on the dendrimer generation [169]. Similarly, the effect of anionic G3.5 and G4 [170] and cationic G4, G5 and G6 [171] PAMAM dendrimers on alpha-synuclein fibril formation was strongly dependent on dendrimer generation, with maximum efficiency observed at maximum dendrimer charge.

**Figure 7:**
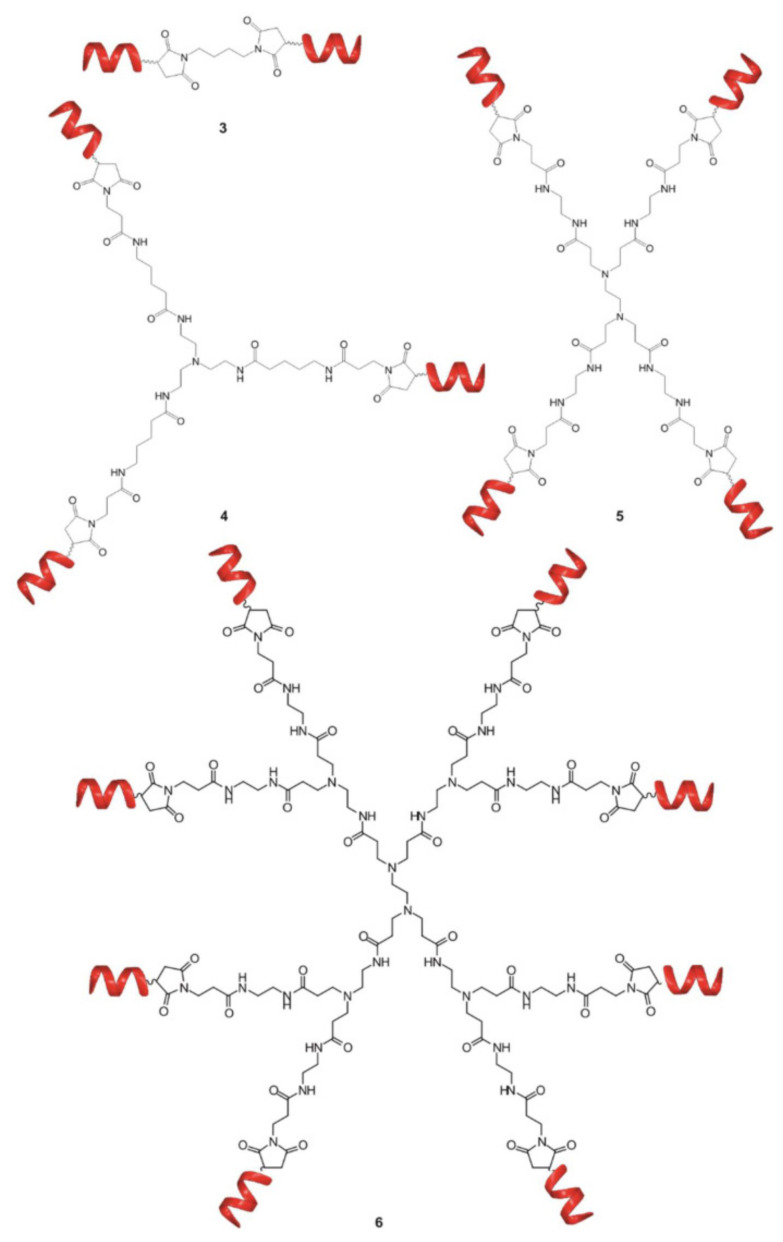
Schematic representation of bivalent (3), trivalent (4), tetravalent (5) and octavalent (6) foldameric conjugates used to study the binding affinities to Aβ oligomers. Reprinted with permission from ref. [160]. Copyright 2018, MDPI.

Notably, the dendritic structure offers tremendous opportunities for controllable modification and modulation of the activity by fine tuning the topology and structure of the molecule. In [160], the authors investigated the influence of the dendrimer branching degree and the chemistry of terminal groups on the ability to interfere with the formation of toxic Aβ oligomers. The results showed that multivalency is crucial for effectively decreasing Aβ oligomers toxicity. For the goal, the di-, tri-, tetra and octa-valent peptide dendritic structures were explored (Figure 7). The association constants measured by ITC demonstrated a clear correlation between binding affinity and valent properties up to tetravalent compounds. Surprisingly, an increase in the number of arms up to eight did not further enhance the affinity of binding, confirming the previous findings that a balance between dendrimer size, charge and charge density is required for effective complexation with protein. The variation of topology from linear to focal symmetry within tetravalent compounds revealed the higher binding affinity for dendritic symmetric compounds. The influence of dendrimer chemistry, i.e., the functional groups, on binding affinity was also assessed through evaluation of thermodynamic parameters obtained by ITC. Replacement of functional groups results in distinct physicochemical properties associated with different binding affinities. Finally, the ability of the selected compounds to decrease the toxicity of Aβ oligomers was studied on hippocampal slices by MTT assay. The results showed a perfect correlation between the binding affinities and biological activity. Similarly, to ITC results, the tetrameric dendrimer was the most efficient in protection against Aβ-induced toxicity, while the octavalent compound did not possess better activity. These outcomes demonstrate that biological activity of the compounds can be predictable from binding data and the dendritic structures are the most potent compounds for the purpose.

The modification of the surface also has a great impact on the ability of dendrimers to interfere with amyloid fibril formation and may vary the mechanism of dendrimer action. Wang et al. [159,161] proposed the hydrophobic binding–electrostatic repulsion (HyBER) theory as a new model for dendrimer binding with amyloidogenic protein for mixed negative charges and hydrophobic groups located at the dendrimer periphery. The authors investigated carboxyl-terminated PAMAM dendrimers of generations 3 to 6 with imbedded phenyl moieties in the inhibition of Aβ42 fibrillation and showed that high generations of phenyl derivatives of dendrimers significantly inhibit the Aβ42 aggregation and alter the ultrastructure of Aβ42 aggregates. Despite the dendrimer binding, unmodified G5 and G6 PAMAM dendrimers were unable to interfere with Aβ fibrillation, while phenyl derivatives G5-P and G6-P demonstrated a high efficiency. Considering the noticeable contribution of entropy in binding, the authors concluded that hydrophobic interactions are responsible for the binding, whereas electrostatic interactions are unfavorable. The results support the previous finding indicating the key importance of hydrophobic interactions for antiamyloidogenic properties of dendrimers.

**Figure 8:**
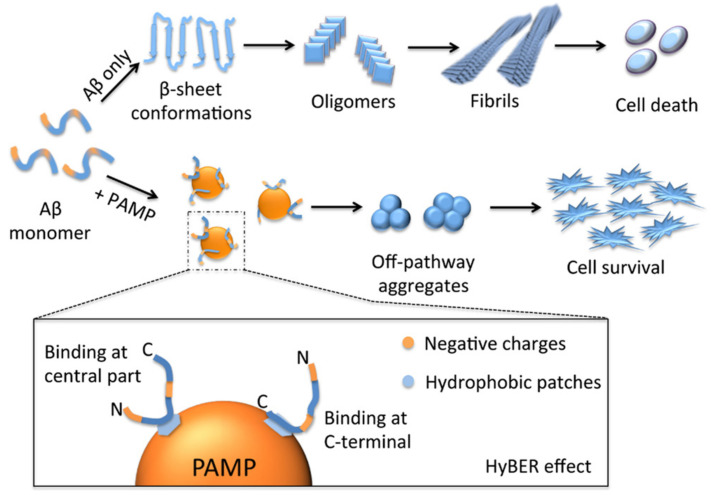
Schematic representation of the inhibition of amyloid aggregation by PAMP caused by the HyBER effect. The top panel represents the on-pathway fibrillation of Aβ42, and the bottom panel represents the off-pathway aggregation of Aβ42 modulated by the PAMP of a proper DS value (e.g., PAMP3 with a DS of 30.5%) by the HyBER effect (depicted in the amplified drawing of Aβ42−PAMP interactions). Reprinted with permission from ref. [159]. Copyright 2018, American Chemical Society.

The studies were further extended and the authors explored the influence of the degree of substitution (DS) by phenyl groups within a single dendrimer generation on the Aβ42 fibrillation [159]. Here, the dendrimers were used as a convenient model for construction of a well-defined structure to explore the applicability and better understand the HyBER theory previously developed. For the goal, G5 PAMAM dendrimer with terminal carboxyl groups was partially substituted by hydrophobic phenyl groups to produce a series of phenyl-derivatized dendrimers (PAMPs) with increasing DS. The effectiveness of inhibition was strongly dependent on the DS of carboxyl surface groups by phenyl moieties, as was confirmed by Thioflavin T (ThT) fluorescence assay. The ThT fluorescence is usually used to monitor the formation of amyloid fibrils since its fluorescence intensity strongly increases in the presence of β-sheet structures. Contrary to unmodified carboxyl-terminated PAMAM dendrimers, an obvious decrease in ThT fluorescence intensity was observed when Aβ42 was incubated with substituted dendrimers (PAMP) even at low concentrations (Aβ42/PAMP = 1:0.01). The effect was more pronounced for the dendrimers with high DS. AFM and CD results confirmed the DS-dependent effect of inhibition of conformational transition to the β-sheet saturated aggregates by PAMPs, with complete inhibition observed for PAMPs with high DS. Notably, the conversion of carboxyl groups to hydroxyl groups suppresses the ability of dendrimers to prevent amyloid fibril formation, indicating the importance of both electrostatic and hydrophobic interactions for antiamyloid activity. This implies the applicability of HyBER hypothesis, providing new insights into the successful design of antiamyloid agents. Particularly, the hydrophobic groups participate in strong binding of amyloidogenic protein with dendrimer, and thus, the bound protein molecule suffers from electrostatic repulsion by anionic groups. Due to these opposite forces, the protein molecule is compelled to extend the conformation distinctly different from the β-sheet structures. The schematic representation of the process is given in Figure 8.

**Figure 9:**
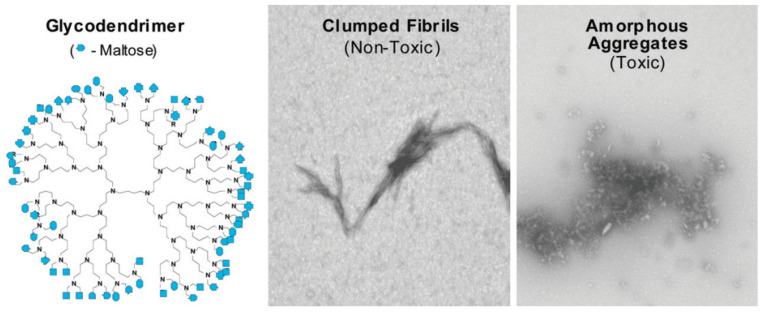
Structure of maltose (Mal)-modified PPI dendrimers and morphology of dendrimer–Aβ(1–40) amyloid aggregates. Transmission electron microscopy micrographs of Aβ(1–40) incubated at pH 7.4 in the presence of PPI-G4-Mal at a dendrimer–peptide ratio of 0.1 (detection of clumped fibrils) and in the presence of PPI-G5-Mal at a dendrimer–peptide ratio of 1 (detection of amorphous aggregates).

The hydrophobic forces significantly contribute to high association constants, which, in turn, influence the ability of dendrimers to induce the conformational changes in protein. In fact, in [157], the authors established the ability to suppress the amyloid aggregation as a result of conformational changes in Aβ42 structure induced by dendrimers. The modification of PPI dendrimers with maltose (Mal) moieties also influenced mechanism of antiamyloid action. While unmodified PPI dendrimers most likely inhibit the aggregation due to binding with monomeric or oligomeric peptide and blocking the fibril growth, PPI-G4-Mal prevent the fibrillation of Aβ42 by shifting the aggregation process to the formation of granular nonfibrillar amorphous aggregates of low toxicity [157]. However, the interaction was generation-dependent: PPI-G5-Mal generated granular nonfibrillar amorphous aggregates, whereas PPI-G4-Mal provided clumped fibrils at low dendrimer−peptide ratios and amorphous aggregates at high ratios (Figure 9). Interestingly, the clumped fibrils were non-toxic for PC12 and SH-SY5Y cell lines, whereas amorphous aggregates demonstrated toxicity. The difference in inhibition pathways was explained by the maltose surface density, which was clearly higher in the case of PPI-G5-Mal. The authors supposed that higher maltose surface density ensures more preferable hydrogen bonding between PPI dendrimers and peptides. 

Similar to prion diseases, the small oligomeric prefibrillar forms of proteins have been found to be the most toxic in another amyloidosis. For that reason, the reports on reducing the content of prefibrillar forms of amyloidogenic proteins are of special interest. For example, in [162], the authors observed a significant decrease in oligomeric forms of Aβ upon the addition of triethylene glycol dendrimer bearing 27 morpholine molecules.

The most recent study exploits all the benefits of dendritic structure combining the inherent antiamyloidogenic properties associated with the high number of terminal groups and their dense packing together with the dendrimer’s cargo capacity. In [172], a hyperbranched polyglycerol (HPG)-based multivalent dendrimer was conjugated with three different antiamyloidogenic small molecules such as gallic acid (GA) [173,174], trehalose (Thl) [175] and tyrosine (Tyr) [28] and tested in the inhibition of protein aggregation. Gallic acid and trehalose are known to have anti-amyloidogenic activity [176], while tyrosine promotes interactions with protein through polar groups and phenolic components [28]. Authors observed an impressive synergetic effect between the antiamyloidogenic action of small molecules and their presence in branched multivalent dendritic structure [172]. While molecular forms of GA, Thl and Tyr are active only at millimolar concentration, conjugated with dendrimers, these molecules are efficient in a micromolar range. The experiments on the HD150Q cell line revealed that functionalized HPG dendrimers effectively hindered intracellular huntingtin aggregation. The GA-modified HPG dendrimer was found to be the most effective.

Recent findings indicate that the intracellular ability of dendrimers to suppress the amyloid toxicity is associated with a pronounced reduction in ROS level in the cell. The viability of the mHippoE-18 cell line with induced alpha-synuclein fibrillation was significantly enhanced in the presence of carbosilane dendrimers [177]. A noticeable decrease in the concentration of rotenone—a molecule responsible for the inhibition of mitochondrial functions and electron transfer during amyloidosis—was observed. Similar results were obtained for PAMAM, phosphorous, viologen–phosphorous and modified HPG dendrimers: a clear decrease in rotenone-induced production of ROS is now recognized as an additional mechanism of the intracellular antiamyloidogenic ability of dendrimers [3,172].

**Figure 10:**
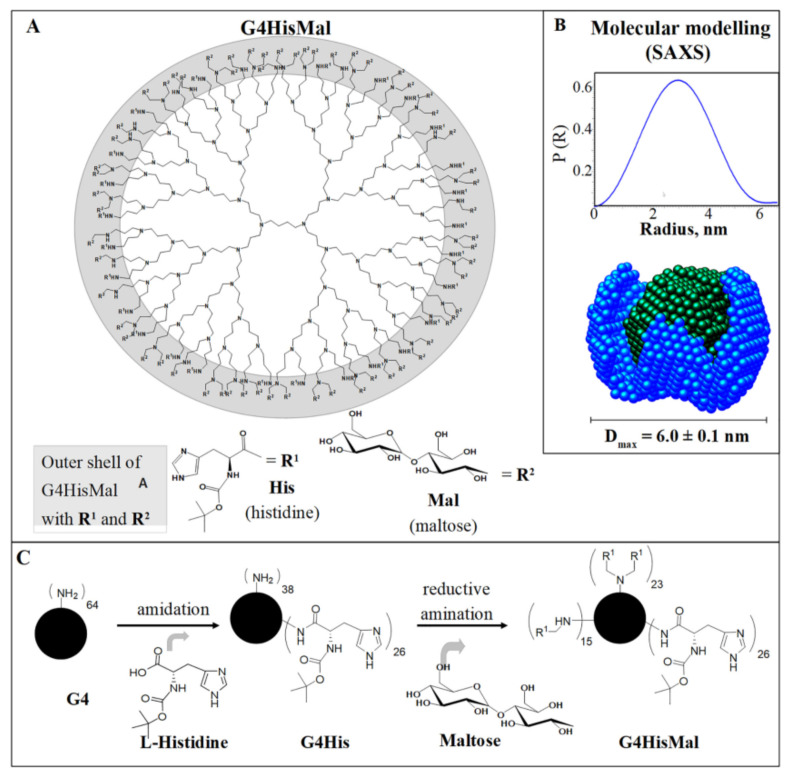
Structure and synthesis of G4HisMal. (**A**) Simplified structure of G4HisMal. Layers 1–4 indicate dendrimer’s branching points and generation of the dendrimer. Layer 4 shows N terminal groups with maltose as R1 and histidine as R2 substituents. (**B**) Distance distribution functions P(R) of G4HisMal in PBS calculated SAXS pattern of G4HisMal in PBS at 37 °C using GNOM.40 Insertion: De novo three-dimensional reconstruction of the scattering entity of G4HisMal using DAMMIF after 10 independent reconstructions. A line indicates the maximum dimension (Dmax). UCSF Chimera41 was used for visualization. (**C**) Reaction pathway for synthesizing glycodendrimer G4HisMal. Reprinted with permission from ref. [178]. Copyright 2019, Elsevier.

Another study [178] demonstrates how the dendrimer structure can be used for the rational design of the molecule with predicted biological activity. Here, the authors constructed the fourth-generation PPI dendrimer with a histidine–maltose shell (Figure 10) (G4HisMal) with the aim to improve the permeability of BBB and further investigate the potential of G4HisMal to protect Alzheimer’s disease-transgenic mice from memory impairment. Histidine is known to selectively pass through BBB and to chelate the Cu ions, which are considered to play an important role in oxidative stress related to Alzheimer’s disease.

**Figure 11:**
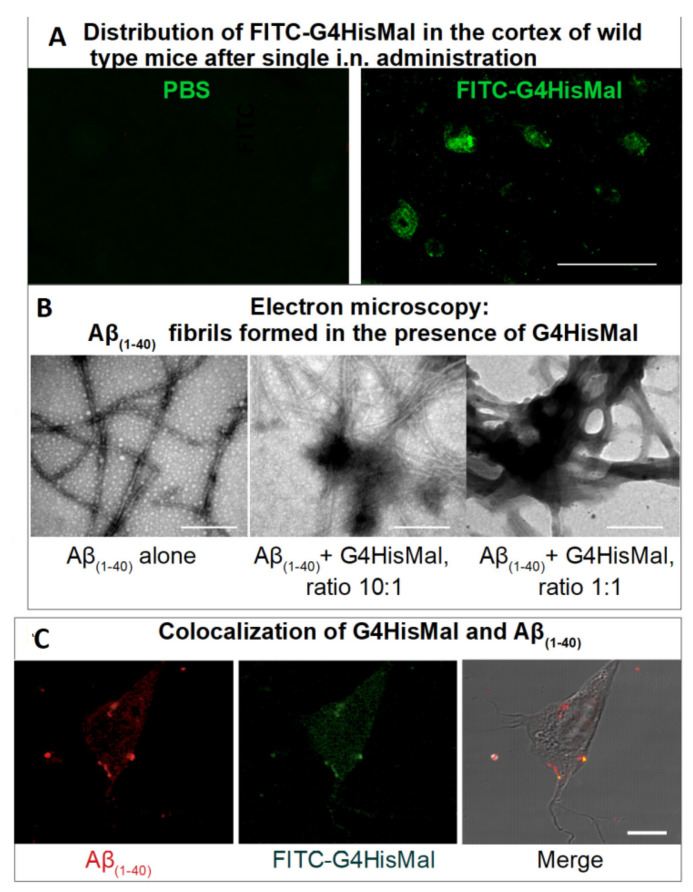
G4HisMal dendrimers cross the BBB and inhibit Aβ_(1–40)_-induced cell toxicity. (**A**) Fluorescence microscopy images show the presence of FITC –G4HisMal in the cortex after i.n. administration of FITC–G4HisMal. The bar is 10 μm. (**B**) Transmission electron microscopy (TEM) of Aβ fibrils. Bar = 100 nm. (**C**) Colocalization of FITC–G4HisMal dendrimers with Aβ_(1–40)_ in SH-SY5Y cells. SH–SY5Y cells were treated with 10 μmol Aβ_(1–40)_ and 1 μmol G4HisMal after 24 h of incubation, then cells were fixed and labeled with specific antibodies against Aβ_(1–40)_ (red) 1 μmol and FITC against FITC-labeled dendrimers (green). Merge shows co-localization of Aβ_(1–40)_ and FITC–G4HisMal. Aβ_(1–40)_ fibrils and monomers were used as controls. Reprinted with permission from ref. [178]. Copyright 2019, Elsevier Ltd.

The authors found that G4HisMal has significantly improved biocompatibility and the ability to cross the BBB. The dendrimers were labeled with fluorescein isothiocyanate (FITC) and administrated intranasally to wild-type mice at a dose of 10 mg/kg (body weight). The presence of G4HisMal in brain was confirmed by immunofluorescent labeling with an antibody specific to FITC (Figure 11A). A comparison of the fluorescence intensities of the brain homogenates treated with G4HisMal and G4Mal (without histidine residues) showed a 40% increase in fluorescence intensity for G4HisMal, which indicated a better penetration ability for histidine-modified dendrimers.

Notably, G4HisMal did not prevent the formation of Aβ_(1–40)_ aggregates but drastically differed their morphology from long, unbranched fibrils to clumped aggregates (Figure 11B). Similarly to previous findings [157], the clumped fibrils were non-toxic for the SH-SY5Y cell line, ranking G4HisMal as a potent compound for the inhibition of Aβ_(1–40)_ cell toxicity: the presence of 1 μmol G4HisMal eliminated the toxicity of 10 μmol Aβ_(1−40)_.

Strikingly, APP/PS1 mice treated with G4HisMal demonstrated a significant memory improvement when compared with the control mice, despite the overall plaque load in the neocortex of treated mice being equal to the control group. The effect was attributed to a reduction in the level and ratio of soluble Aβ oligomers, which was confirmed experimentally.

To clarify the possible mechanism of memory rescue by G4HisMal, the levels of pre- and postsynaptic markers—synaptophysin and Pds95—were identified. The decrease in the level of these markers is now accepted to be responsible for cognitive decline in human Alzheimer’s disease. Remarkably, G4HisMal was able to survive the amount of synaptophysin and Pds95 in treated APP/PSI mice compared to the control group, recognizing this effect as one of the possible mechanisms underlying the synapse and memory protection by dendrimers. The results are promising and open a new avenue for the development of molecules that may protect synapses and fight against memory decline in neurodegenerative disorders.

## 5. Conclusions and Outlook

Dendrimers are often called “nanomaterials of the future” and have already found applications in some fields of medicine. A striking example is the antiviral drug VivaGel (Starpharma, Australia), in which the active substance is the polylysine dendrimer modified with naphthalenedisulfonic acid. Some drugs based on dendrimers exhibiting antitumor activity, for example, DEP docetaxel and DEP cabazitaxel (Starpharma, Australia), are now in clinical trials. Nevertheless, the successful application of dendrimers in medicine is directly connected with the elucidation of the fundamental principles of dendrimers’ interaction with biomolecules, the investigation of structure–activity relationships and a complete physicochemical characterization of the complexes formed. In this regard, the discovery of the principles of dendrimer interaction with amyloidogenic proteins plays an important role in understanding the development of pathologies associated with the conformational transformations of proteins and contributes to the investigation of the mechanisms of control over protein aggregation via exogenic ligands.

The dendritic structure offers several benefits for the development of novel approaches to the treatment of neurodegenerative disorders.

First of all, tight control over the structure of the molecule, based on the controlled variation of certain structure elements and fine tuning of the molecule topology, allows one to study in detail the relationship between structure and activity. This allows directed molecule design with predictable biophysical properties and biological activities and opens new prospects for the synthesis of chemical compounds featuring the antiamyloid activity.

A further dendritic advantage implies the ability to combine the inherent anti-amyloid properties of dendrimers with other known therapeutic options, such as selective targeting or cargo capacity of internal cavities. Simple filling of dendrimers with small molecules with known antiamyloid activity results in a synergistic effect that remarkably enhances efficiency. We expect this line of research to receive wide scientific attention and provide new opportunities for the inhibition of amyloid propagation and modulation of protein–protein interactions. Finally, of particular importance is the established ability of dendrimers to rescue memory impairments observed in mice with a model of Alzheimer’s disease. The discovery certainly proves that dendrimers are able not only to modulate the process of amyloid aggregation, but also improve clinical symptoms in vivo and provide memory protection.

Given that treatments that may protect the synapses are one of the possible ways to combat cognitive decline in neurodegenerative diseases, dendrimers are potential drug candidates for synapse protection. Further research should be focused on the elucidation of the mechanism that defines the memory protection ability and on verifying the results in other models.
